# Analysis of the mechanisms and efficiency of Taxifolin encapsulation in whey proteins via thermomechanical mixing and spray drying

**DOI:** 10.1016/j.fochms.2025.100261

**Published:** 2025-05-27

**Authors:** Aleksander A. Borisenko, Tatyana N. Bobrysheva, Marina S. Zolotoreva, Georgiy S. Anisimov, Aleksey A. Borisenko, Dmitry G. Alexeev, Victoria G. Razinkova, Ekaterina G. Greseva, Svetlana S. Avanesyan, Marina N. Sizonenko, Natasa Poklar Ulrih, Itai S. Dzinamarira

**Affiliations:** a Faculty of Food Engineering and Biotechnology named after Academician A.G. Khramtsov, North-Caucasus Federal University, Stavropol 355017, Russia; b Biotechnical Faculty, University of Ljubljana, Ljubljana 1000, Slovenia; c Amrita School for Sustainable Futures, Amrita Vishwa Vidyapeetham, Amrita University, Amritapuri Campus, Kollam, Kerala 690525, India; d Faculty of Commerce, Zimbabwe Open University, Manicaland Regional Campus, Stand 92, C Avenue, Mutare Box Address, P.O. Box V7480, Zimbabwe

**Keywords:** Polyphenols, *Α*-Lactalbumin, *Β*-Lactoglobulin, Molecular modeling, Microcapsules, Taxifolin crystals, Antioxidant activity

## Abstract

Taxifolin (TXL) is a minor bioactive compound from the polyphenol class that may significantly impact human metabolism when included in food products. However, its application is limited by a bitter taste and low bioavailability. We hypothesized that encapsulating TXL in whey proteins using thermomechanical mixing or spray drying could effectively mask its bitterness and enhance bioavailability. Computational simulations indicated that each molecule of β-lactoglobulin (β-Lg) and α-lactalbumin (α-La) can bind at least one TXL molecule. Consequently, experiments used an equimolar ratio of whey proteins to TXL (1:1). Thermomechanical stirring of liquid whey protein concentrate (WPC) at 80 ± 2 °C followed by lyophilisation formed protein aggregates up to 160 μm in size, incorporating large TXL crystals. Encapsulation efficiency was 63 ± 3 %, and the bitter flavour remained unmasked. In contrast, encapsulation via spray drying achieved up to 71 ± 2 % efficiency at an inlet air temperature of 150 °C. The resulting WPC microcapsules, up to 30 μm in size, did not contain large TXL crystals, and bitterness was fully masked. This aligns with the observed reduction in TXL's antioxidant activity. After disrupting WPC microcapsules with ethanol, antioxidant activity of the polyphenol was nearly fully restored. These findings suggest that when such microcapsules are ingested with food, the antioxidant activity of TXL will be expressed in the intestine following proteins breakdown. The results may support the development of novel food products containing whey proteins with encapsulated TXL.

## Highlights

• Computational models of whey proteins and taxifolin (TXL) complexes were obtained.
• Equimolar ratio of components was established when encapsulating TXL in whey protein concentrate.
• TXL bitterness was not masked by thermomechanical mixing and freeze-drying.
• Spray drying at 150 °C masked TXL bitterness.
• TXL antioxidant activity was restored when microcapsules were disrupted by ethanol.

## Graphical abstract

**Figure:**
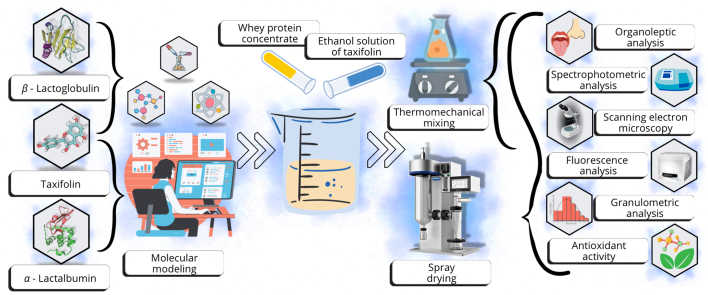

## 1 Introduction

Taxifolin (dihydroquercetin) is a representative natural compound from the polyphenol class, belonging to the flavonoid group (Bobrysheva et al., 2023; Fatkullin et al., 2023; Singla et al., 2019; Zhang et al., 2021). In contemporary nutritional science, polyphenols are classified as minor bioactive compounds. They play a significant role in human metabolism and must be included in the nutriome, which refers to the formulation of optimal nutrition (Tutelyan et al., 2020). Polyphenols, including TXL, are among the most extensively studied and safest compounds (Das et al., 2021; Liu et al., 2023; Turck et al., 2017), renowned for their broad spectrum of biological activities. As a result, they have long been of considerable interest to the scientific community as potential agents for the enrichment of food products (Bobrysheva et al., 2023; Fujitaka et al., 2019).

The primary focus on polyphenols stems from their interaction with antioxidant mechanisms in the human body, which is attributed to the presence of multiple hydroxyl groups in their molecular structure (Singla et al., 2019; Tang et al., 2019). This has been convincingly demonstrated by numerous studies (Choi et al., 2009; Fraga et al., 2019; Ma et al., 2018; Ying et al., 2021).

The beneficial effects of polyphenols on human health may also stem from their impact on the gut microbiota (Istas et al., 2019; Mompeo et al., 2020; Ntemiri et al., 2020; Tan et al., 2024). It has been established that the inclusion of TXL in the diet of mice exerts an inhibitory effect on the overgrowth of proteobacteria induced by an obesity-promoting diet and, in general, improves the diversity of the gut microbiota (Su et al., 2022).

The diverse range of favourable physiological effects exerted by polyphenols has generated considerable interest in these compounds as valuable dietary ingredients. However, polyphenols are typically present in small quantities in most natural sources and are concentrated in plant by-products, such as oilcake from olive oil production, grape skins and seeds from winemaking, and citrus peel and membranes from juice production (Caballero et al., 2022). Polyphenol-rich foods often possess a characteristic tart or astringent taste – such as strong tea or coffee, bitter chocolate, citrus fruits, and persimmons. These organoleptic properties can limit the potential for artificially enriching products with phenolic compounds. Free polyphenols, by interacting with salivary mucin, diminish its ‘shielding’ effect on taste receptors, thereby intensifying the perception of astringency. Furthermore, polyphenols are known to exhibit poor absorption in the gastrointestinal tract in their natural form. Their low bioavailability is largely attributed to their poor solubility and chemical instability (Crespy et al., 1999; Fatkullin et al., 2023; Hu et al., 2003; Marín et al., 2015).

These challenges underscore the need to explore technological solutions for creating polyphenol-enriched products that can deliver them to the targeted areas of the gastrointestinal tract. One such solution is encapsulation, which is increasingly employed in the development of food products and delivery systems for bioactive substances within the food industry (Caballero et al., 2022). Encapsulation not only masks the undesirable taste of polyphenols but also enhances their stability against external factors (Maqsoudlou et al., 2020; Maqsoudlou et al., 2022), preserves their beneficial physiological effects, and improves their bioavailability (Caballero et al., 2022; Choi & McClements, 2020; Fatkullin et al., 2023; Steiner et al., 2019).

Both natural and synthetic biodegradable high-molecular-weight compounds serve as encapsulating materials, with various proteins being widely utilised (Bilia et al., 2014).

Whey proteins, in particular, are recognised for their excellent technological properties and nutritional value (De Wit, 1998; Khramtsov et al., 2021). They can interact with polyphenols to improve their stability during metabolic breakdown under digestive conditions, thereby enhancing their antioxidant activity (Chavoshpour-Natanzi & Sahihi, 2019; Cutrim & Cortez, 2018; Kanakis et al., 2011; Wang et al., 2015). This highlights the importance of continued research into the use of whey proteins in encapsulation technology.

Thermomechanical mixing and spray drying are among the most straightforward methods for implementing the encapsulation process in the food industry. Thermomechanical mixing equipment is extensively used in the production of a wide range of food products, enabling reduced energy consumption by integrating multiple technological processes (Zhao et al., 2011). Spray drying systems, renowned for their high efficiency and capacity, are currently the most widely employed equipment for producing a diverse array of dry food products and ingredients (Banjare et al., 2019; Castro-López et al., 2021). We hypothesized that encapsulating TXL in whey proteins using thermomechanical mixing or spray drying could effectively mask the bitter taste of the polyphenol and enhance its bioavailability.

The aim of this study was to assess the efficiency and identify the optimal process conditions for encapsulating taxifolin (TXL) into whey proteins using thermomechanical mixing and spray drying. Concurrently, experimental investigations were conducted based on molecular predictions regarding the mechanisms and characteristics of the interaction between the polyphenol and the primary whey protein fractions.

## 2 Materials and methods

### 2.1 Materials

Taxifolin (TXL) in powder form with a purity of 98.5 % was supplied by Ametis JSC (Blagoveshchensk, Russia) for the study. For encapsulation, liquid whey protein concentrate (WPC) was used, produced by Stavropolsky Dairy Plant JSC (Stavropol, Russia). The mass fraction of dry matter in WPC was 21.89 ± 1.43 %, with an active acidity of pH 6.36 ± 0.05, and a protein fraction of 50 %. All reagents used were of analytical grade, including: Folin-Ciocalteu reagent (Scharlab S.L., Barcelona, Spain), sodium carbonate decahydrate, sodium hydroxide (NevaReaktiv, Saint-Petersburg, Russia), 1 % alcoholic phenolphthalein solution, cobalt sulfate (Chemical Point UG, Deisenhofen, Germany), sodium persulfate (Sisco Research Laboratories, Mumbai, India), sodium dihydrogen phosphate, sodium hydrogen phosphate (Lumex, Saint-Petersburg, Russia), ABTS radical cation (2,2′-azinobis-3-ethylbenzthiazoline-6-sulfonic acid) (Applichem, Darmstadt, Germany). The samples were prepared using deionized water obtained from the water purification system HLP 5UV (Hydrolab, Straszyn, Poland).

### 2.2 Molecular modeling

The study of the mechanisms of interaction between whey protein molecules and TXL, as well as conformational analysis, calculations, and prediction of molecular system characteristics, was carried out using molecular mechanics methods MM+ and AMBER, the semi-empirical CNDO method, and molecular dynamics in the computer programs HyperChem, NAMD, and VMD (USA) (HyperChem, https://www.hypercubeusa.com, accessed on 23 August 2024; Phillips et al., 2020; Humphrey et al., 1996). The models of the tertiary structure of whey protein molecules were constructed using resources from the international protein and nucleic acid spatial structure database, Protein Data Bank (wwPDB, http://www.wwpdb.org, PDB code 3NPO, 1HFZ, accessed on 19 July 2024).

### 2.3 TXL encapsulation

The TXL was initially dissolved in 96 % ethanol (140 mg/mL). The resulting TXL solution was then added in an equimolar ratio (1:1) to the liquid WPC and stirred using a magnetic stirrer (MSH-300i digital, BioSan, Riga, Latvia) for 5 min at 500 rpm.

Two encapsulation methods were employed: thermomechanical mixing and spray drying.

#### 2.3.1 Thermomechanical mixing

Thermomechanical mixing was used to perform the encapsulation by stirring the WPC solution containing TXL in equimolar ratio (1:1) on a magnetic stirrer for 20 min at 1000 rpm, with heating to 80 ± 2 °C. It is well-established that this temperature induces active denaturation of all whey protein fractions (Hollar et al., 1995). Subsequently, the samples were frozen at −25 °C for 72 h, subjected to lyophilisation using an LS-500 lyophiliser (Prointech, Saint-Petersburg, Russia), and then ground into a fine powder using a laboratory planetary mill (Pulverisette 5, Fritsch, Idar-Oberstein, Germany).

#### 2.3.2 Spray drying

Spray drying was conducted using a laboratory-scale unit (Mini Spray Dryer B-290, Buchi, Flawil, Switzerland) with an evaporation capacity of 1 L/h. The inlet air temperatures were set at 130, 150, and 170 °C, while the corresponding outlet air temperatures were 70, 73, and 75 °C, respectively. The drying solution was delivered through a standard dual-flow nozzle with an internal diameter of 7 mm. Compressed air was supplied at a pressure of 6 bar, flow rate was 82.5 m^3^/h.

### 2.4 Determination of encapsulation efficiency by Folin-Ciocalteu assay

The encapsulation efficiency of TXL was assessed by determining the free polyphenol content using the Folin-Ciocalteu method (Pérez et al., 2023). A 200 mg aliquot of dry WPC containing encapsulated TXL was mixed with 5 mL of a 60 % aqueous ethanol solution, stirred, and centrifuged for 10 min at 13,000 rpm. The supernatant was then collected for analysis. In a clean test tube, 0.2 mL of supernatant was placed, followed by the addition of 0.45 mL of distilled water. Then, 0.1 mL of Folin-Ciocalteu reagent was added, mixed, and left for 3–5 min. Afterward, 0.4 mL of a 7.5 % sodium carbonate solution was added. The mixture was stirred and left in the dark for 60 min. The optical density was measured using a Unico 2800 single-beam scanning spectrophotometer (United Products and Instruments, Dayton, NJ, USA) with quartz cuvettes at a wavelength of 765 nm. A control sample was prepared under identical conditions using pure dry WPC to exclude the influence of whey proteins on the optical density of the solutions. The difference between the readings of the experimental and control samples was used for calculations. The free TXL content was determined from the calibration curve, and the amount of encapsulated polyphenol was calculated as the difference between the total TXL (*W*_T_) incorporated into the sample and its free form (*W*_F_). Encapsulation efficiency (*EE*) was expressed as the percentage of the total TXL incorporated into the sample:

(1) $$\mathrm{EE}=\frac{\left(W_{T}–W_{F}\right)}{W_{F}}\times 100\%$$

### 2.5 Scanning electron microscopy

Scanning electron microscopy was performed using a Tescan Mira 3 LMH microscope (Tescan, Brno, Czech Republic) in standard secondary electron imaging mode, with an accelerating voltage of 10 kV.

### 2.6 Fluorescence emission spectrometry

For analysis, dry samples of pure WPC and WPC with encapsulated TXL were pre-dissolved. To a 200 mg suspension, 4 mL of phosphate buffer (pH 6.5) was added, corresponding to the normal acidity of WPC. A 250-fold dilution was then performed. A separate sample was prepared to evaluate the effect of unbound taxifolin on changes in fluorescence intensity. In this case, TXL was added to the WPC solution in an equimolar ratio (1:1) immediately during sample preparation. A 100 μL aliquot of each solution was transferred into the wells of a 384-well black microplate. Fluorescence emission intensity was measured at an excitation wavelength of 280 nm, with emission recorded from 300 to 420 nm in 5 nm increments (Acharya et al., 2013; Hemar et al., 2011). The analysis was conducted using a Varioscan LUX fluorimeter (Thermo FS, Waltham, MA, USA) at 20 °C. The fluorescence emission intensity of the TXL-containing samples was expressed as a percentage of the fluorescence intensity of pure WPC.

### 2.7 Granulometric analysis

The particle size distribution of the WPC microcapsules was measured using a Shimadzu SALD-7500 analyzer (Shimadzu, Kyoto, Japan) via laser diffraction, in accordance with ISO 13320:2020. A SALD-MS75 flow cell was employed for the analysis, ensuring continuous circulation of the sample and mitigating the impact of sedimentation on the measurement results. The results were processed using WingSALD II software.

### 2.8 Antioxidant activity

The antioxidant activity of TXL was assessed using the ABTS assay (Re et al., 1999). Samples were dissolved in 5 mL of a 60 % aqueous ethanol solution and placed in 1.5 mL microcentrifuge tubes, followed by centrifugation at 13,000 rpm for 15 min (MicroCl 17R, Thermo FS, Waltham, MA, USA). The ABTS^●+^ cation radical was generated by incubating a mixture (1:1) of 7 mM ABTS and 2.45 mM potassium persulfate solution. The mixture was kept in the dark at room temperature for 12–16 h. Prior to the analysis, the ABTS^●+^ solution was diluted with ethanol to achieve an optical density of 0.70 ± 0.02 at 734 nm. The analysis was performed using a SF-102 spectrophotometer (Aquilon, Podolsk, Russia). In a cuvette with a 1 cm path length, 1.98 mL of ABTS^●+^ solution was placed, followed by the addition of 0.02 mL of the sample solution. The optical density was recorded after 3 min. The inhibition of ABTS^●+^ was calculated as the percentage reduction in absorbance. Antioxidant activity was expressed as trolox equivalent antioxidant capacity (TEAC) units based on a standardized calibration curve with trolox (Re et al., 1999).

### 2.9 Statistical processing

All experimental studies were conducted under identical conditions. Statistical analysis was performed using Microsoft Excel 2019, supplemented with XLSTAT statistical software. Results are presented as the mean ± standard deviation (SD) for samples collected from three independent runs. The reliability of the experimental data for all parameters was assessed using Student's *t*-test with a significance level of *p* ≤ 0.05 based on three technical replicates.

## 3 Results and discussion

### 3.1 Modeling the mechanisms of interaction between taxifolin molecules and whey proteins

Polyphenols exhibit potent biological activity and can produce beneficial physiological effects in the human body (Fujitaka et al., 2019; Mompeo et al., 2020). It is well established that they can form complexes with various proteins. Consequently, understanding the mechanisms of interaction between polyphenol molecules and proteins is of significant interest for the development of fortified food products (Shahidi & Dissanayaka, 2023).

Existing studies (Geng et al., 2020; Kanakis et al., 2011) indicate that milk proteins possess the capacity to bind polyphenols. The mechanisms of interaction between proteins and polyphenols can vary depending on the protein's structural properties and the conditions of the dispersion medium. These interactions may include the formation of hydrogen bonds, hydrophobic interactions between the aliphatic and aromatic fragments of the protein and the aromatic rings of polyphenol molecules, as well as electrostatic interactions. The relative contribution of each interaction type depends on the specific structural characteristics of both the polyphenol and the protein (Kanakis et al., 2011).

Modeling and predicting the mechanisms of interaction between polyphenols and whey proteins is of considerable scientific and practical interest, particularly for evaluating the feasibility and efficiency of polyphenol encapsulation. At this stage, it was essential to determine the complementarity between the molecules of key whey proteins (*β*-lactoglobulin, *β*-Lg, and *α*-lactalbumin, *α*-La) and taxifolin (TXL). This complementarity is defined by the mutual correspondence of their molecular surfaces, which facilitates the formation of bonds through various intermolecular interactions.

#### 3.1.1 Structural models of β-Lg, α-La and TXL molecules

In whey, approximately 58 % of all proteins are *β*-lactoglobulin (*β*-Lg) (Madureira, Pereira, Gomes, Pintado, & Xavier Malcata, 2007). In its native state, *β*-Lg (with a pH range of 5.3–6.7) typically exists as a dimer with a subunit molecular mass of approximately 18,350 Da. Each monomer consists of 162 amino acids, one free cysteine, and two disulfide bridges (Kontopidis et al., 2004).

**Fig. 1:**
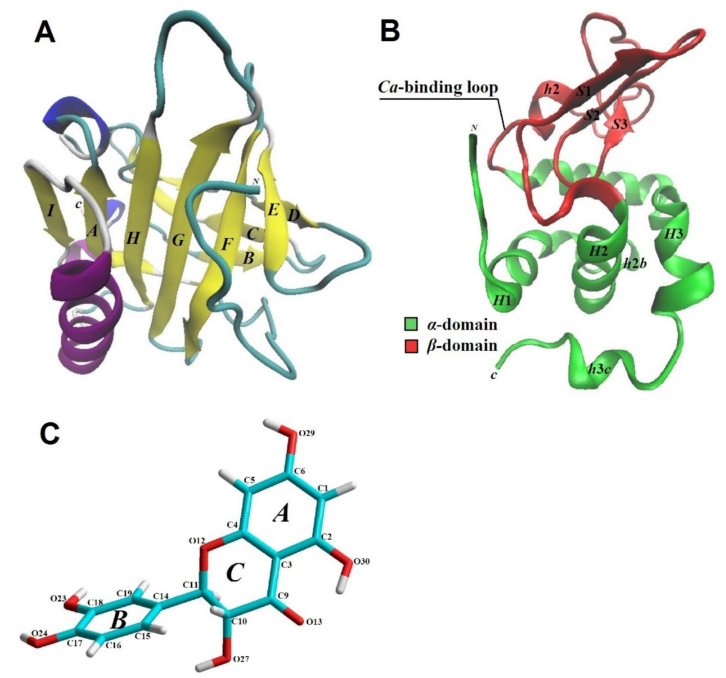
Models of the tertiary structure of whey protein molecules *β*-Lg (A), *α*-La (B) and the spatial structure of the TXL molecule (C).

*β*-Lg is classified as a prototypical lipocalin. The structure of its molecule (Fig. 1A) consists of nine antiparallel *β*-sheets and one *α*-helix, with the *β-*sheets comprising 43 %, the *α*-helix 10 %, and disordered regions, including *β*-loops, accounting for 47 % of the total structure (Brownlow et al., 1997; Kontopidis et al., 2004).

The eight *β*-sheets (designated *A-H)* form a *β*-barrel, the interior of which contains hydrophobic amino acid residues. Sheets *A-D* constitute one side of the barrel, while sheets *E-H* form the opposing side. An *α*-helix is positioned on the outer surface of the barrel, between *β*-sheets *A* and *H*.

There is compelling evidence that *β*-Lg, like many lipocalins, is capable of binding small hydrophobic and amphiphilic molecules, including hexane, palmitic acid, vitamin D, and cholesterol (Loch et al., 2012; Yang et al., 2008). It has been definitively established that *β*-Lg can bind retinol (Newcomer et al., 1984). Notably, studies indicate that the central barrel of *β*-Lg serves as the major binding site for such ligands (Kontopidis et al., 2004; Wu et al., 1999).

In terms of quantitative content, the second most abundant protein in whey is *α*-lactalbumin (*α*-La), which accounts for approximately 20–25 % of total whey protein content (Madureira et al., 2007). *α*-La is a globular calcium-binding protein with a molecular mass of approximately 14,175 Da and an isoelectric point at pH 4.6 (Fiorucci et al., 2007; Wright et al., 2001). It shares a close evolutionary relationship with *C*-type lysozymes in both its primary and tertiary structure (Rice-Evans, 2012).

The native structure of *α*-La is characterised by two primary domains: a large *α*-helical domain and a smaller *β*-domain, connected by a calcium-binding loop (Fig. 1B). The big *α*-domain consists of three main helices (*H1−H3*), along with two short *α*-helices (*h*1*b* and *h*3*c*), spanning amino acid residues 1–34 and 86–123. The smaller *β-*domain, which spans residues 35–85, includes a series of loops, three antiparallel *β-*sheets (*S*1-*S*3), and a short *α*-helix (*h2*).

The domains are separated by a deep cleft and held together by a disulfide bridge between the amino acid residues *Cys73* and *Cys91*. Overall, *α*-La's structure is stabilized by four disulfide bridges, formed between residues 6–120, 61–77, 73–91, and 28–111.

It is well established that *α*-La can bind calcium ions, which are crucial for stabilizing its tertiary structure (Rice-Evans, 2012). The calcium ion is coordinated in a loop between the *α*- and *β-*domains, where it is surrounded by five oxygen-containing groups from the residues *Lys79, Asp82, Asp84, Asp87*, and *Asp88*, along with additional coordination from one or two water molecules.

Flavonoids, including TXL, are classified as C6-C3-C6 compounds, with their molecular structure consisting of two benzene rings (*A* and *B*) linked by a three‑carbon fragment. The heterocyclic ring of the TXL molecule adopts an asymmetric armchair conformation with a planar system of four oxygen (O12)‑carbon (C4, C3, C9) atoms.

In our constructed spatial model of the TXL molecule (Fig. 1C), the torsional angle around the O12-C11-C14-C19 bond is 78.33°, which is consistent with available studies of its crystal structure (78.4°) (Selivanova et al., 1999).

In different flavonoid compounds, the position of the substituted phenyl ring relative to the heterocyclic system varies from almost parallel to almost perpendicular (Koroteev et al., 2014), which is attributed to the low barrier of rotation of the *B* ring around the ordinal bond, as well as to the different environment of the molecules.

#### 3.1.2 Prediction of interactions between TXL and β-Lg molecules

The analysis of the *β*-Lg molecular model revealed an absence of distinct centres exhibiting either an excess or deficit of electron density on its surface. The central barrel of *β*-Lg is characterised by an electrostatic potential value close to zero and represents its main hydrophobic cluster. Examining this hydrophobic cluster allows for the suggestion of the functional roles played by various amino acid residues in the formation of protein-ligand complexes. Additionally, this analysis supports theoretical predictions of the mechanisms underlying their intermolecular interactions (Karyagina et al., 2006).

As a result of the conducted modeling, the primary potential binding site of the *β*-Lg molecule for the TXL molecule has been identified*,* comprising the amino acid residues of hydrophobic barrel *Val41*, *Val43*, *Ile56*, *Ile71*, *Met107*, and *Phe105*, with possible involvement of Pro38, *Lys60, Lys69,* and *Gln120*. The spatial arrangement and the composition of the functional groups of these amino acid residues are critical not only for the folding and stabilization of the *β-*Lg structure but also in its interaction with ligands (Chavoshpour-Natanzi & Sahihi, 2019).

**Fig. 2:**
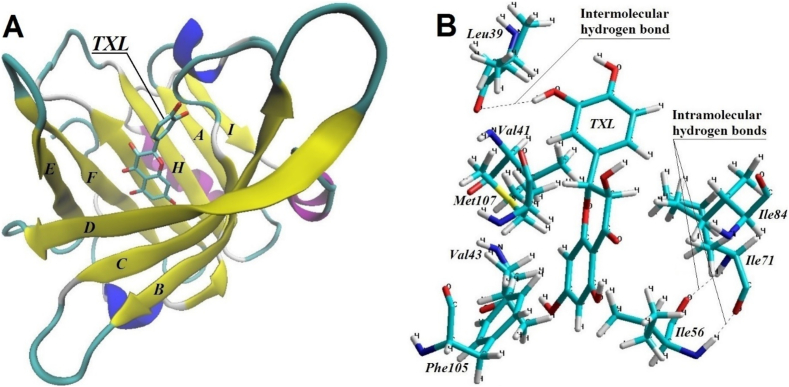
A model of the *β*-Lg-TXL molecular system (A). The mutual arrangement model of the amino acid residues within the hydrophobic barrel of the *β*-Lg molecule and the TXL molecule during their interaction (B).

Under simulated aqueous conditions (water box), a TXL molecule was positioned within the hydrophobic barrel region of the *β*-Lg molecule, and the entire system was optimized to determine its energetically favourable state. Conformational analysis revealed persistent hydrophobic interactions between the components of the system throughout the depth of the *β*-Lg hydrophobic barrel. This interaction is characterised by the movement of nonpolar regions of the molecules towards each other, reducing the number of surrounding water molecules. Consequently, the nonpolar amino acid residues *Val41*, *Val43*, *Ile56*, *Ile71*, *Ile84, Phe105, and Met107* of the protein were closely aligned with the aromatic rings of the TXL molecule (Fig. 2).

Upon interaction with the *β*-Lg molecule in an aqueous environment, the torsional angle around the O12-C11-C14-C19 bond of the TXL molecule increased from 78.33° to 88.00° which can be attributed to the formation of intermolecular bonds. The change in the torsional angle between the phenyl ring and the heterocyclic system is the most likely consequence of polyphenol interactions with surrounding molecules (Koroteev et al., 2014).

The average distance between the nonpolar side chains of amino acid residues *Val41*, *Ile71*, *Ile84, and Met107,* and the TXL molecule's benzene core *B* and three‑carbon fragment, which are involved in the hydrophobic interaction, was calculated to be 2.832 Å. The benzene core *A* of the TXL molecule formed stable hydrophobic interactions, positioned nearly equidistant between the alkyl side chain of *Val43*, the aromatic ring of *Phe105*, and the hydrocarbon side chain of *Ile56* (Fig. 2B). The average distance between the atoms of the benzene core *A* and the nonpolar side chains of these amino acid residues was found to be 2.877 Å. Although not all apolar amino acid residues from the central barrel of the *β*-Lg molecule participate in forming hydrophobic interactions with the TXL molecule, our results suggest that hydrophobic interaction plays a significant role in the formation of the β-La-TXL molecular complex.

As a result of the molecular-level analysis, it was also established that the amino acid residue *Leu39*, located in the *β*-loop region near the neck of the hydrophobic barrel between sheets A and B, participates in the interaction with the TXL molecule. A hydrogen bond was identified between the hydrogen atom of the hydroxyl group on the benzene core *B* of the TXL molecule and the oxygen atom of the carbonyl group of *Leu39*. Hydrogen bonds are one of the key factors in the stable binding of polyphenols to proteins (Feng et al., 2023). The polyphenol molecule serves as a hydrogen atom donor, with its hydroxyl groups capable of forming hydrogen bonds with oxygen or nitrogen atoms in the side chains of amino acid residues. Each hydrogen bond formed through this interaction contributes significantly to the stabilization of the intermolecular complex (Shahidi & Dissanayaka, 2023).

Using the semiempirical quantum-chemical CNDO method, the electronic characteristics of the *β*-Lg fragment involved in the interaction with the TXL molecule were computed. The total energy of the system decreased by 153,643.1 kcal/mol as a result of this interaction, indicating the formation of a stable bond between the molecules.

Therefore, the placement of the TXL molecule within the hydrophobic barrel of the *β*-Lg molecule, along with their size compatibility, the specific nature of the hydrophobic interactions, and the energetically favourable configuration of the formed complex, suggests a high degree of complementarity. This supports the potential for efficient encapsulation of the polyphenol within the protein matrix.

#### 3.1.3 Prediction of interactions between TXL and α-La molecules

**Fig. 3:**
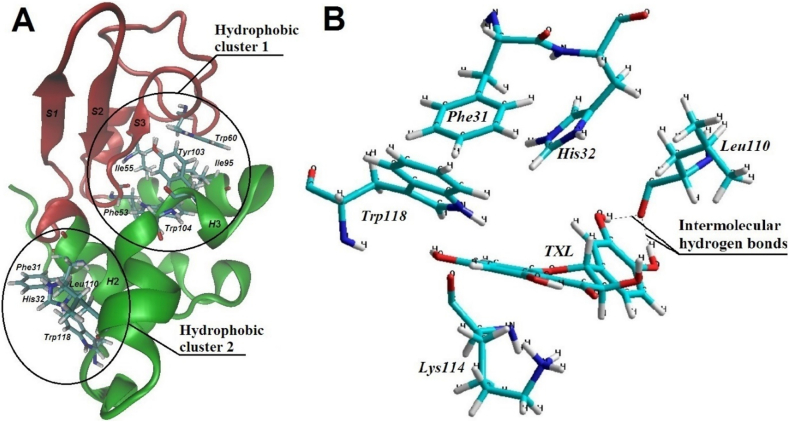
Model of the tertiary structure of the *α*-La molecule with hydrophobic clusters (A). Model of the mutual arrangement of nonpolar amino acid residues of hydrophobic cluster 2 of *α*-LA molecule and TXL molecule during their interaction (B).

The analysis of the *α*-La molecular model indicates that, in contrast to the *β*-Lg molecule, its surface exhibits a heterogeneous electrostatic potential characterised by distinct regions of both electron density excess and deficiency. On the surface of the *α*-La molecule, two primary hydrophobic clusters have been identified, which are of particular interest regarding potential interactions with polyphenol molecules (Fig. 3A).

Both hydrophobic clusters are primarily aromatic in nature and are located within the key functional regions of the *α*-La molecule (Pike et al., 1996).

The first hydrophobic cluster, located in the cleft between the two domains of the *α*-La molecule, represents its hydrophobic core and is composed of the amino acid residues *Phe53*, *Ile55*, Trp60, *Ile95, Tyr103*, and *Trp104.* The second cluster consists of the amino acid residues *Phe31 and His32*, located at the end of the *H*2 helix, directly adjacent to the lower limits of the cleft, the amino acid residue *Leu110*, situated at the end of the *H*3 helix, and the amino acid residue *Trp118*, located at the *C*-terminal tail of the *α*-La molecule.

In the subsequent step, a TXL molecule was alternately placed within the hydrophobic cluster regions of the *α*-La molecule, and the entire system was optimized to determine its energetically favourable state.

Conformational analysis revealed that stable interaction between a single *α*-La molecule and the polyphenol in the region of hydrophobic cluster 1 was unlikely. This is attributed to the dense packing of hydrophobic groups of the protein's amino acid residues within the cluster. The cleft of the *α*-La molecule, where the hydrophobic cluster is located, does not have a sufficient size to accommodate the entire TXL molecule. As a result, a significant portion of the nonpolar groups within the cluster remains inaccessible for intermolecular interactions.

Consequently, in this region, the *α*-La-TXL system lacks the necessary complementarity to facilitate a stable interaction.

In contrast, hydrophobic cluster 2 forms a small depression on the surface of the *α*-La molecule and exhibits high accessibility for intermolecular interaction with the polyphenol. The surface of the TXL molecule matches the geometry of this depression with sufficient precision to enable interaction with the amino acid residues forming the cluster, thereby positioning the TXL molecule stably within it.

Further conformational analysis confirmed the potential for interaction between the molecules in this region. The movement of the nonpolar parts of the molecules towards each other, accompanied by a reduction in surrounding water molecules, was observed. As a result, the amino acid residues *Phe31, His32, Leu110, and Trp118* of the protein, along with the aromatic rings of the TXL molecule, were positioned in close proximity to one another (Fig. 3B).

The torsional angle around the O12-C11-C14-C19 bond of the TXL molecule, upon interaction with the *α*-La molecule in the aqueous environment, was 63.90°. The average distance between the nonpolar radicals of amino acid residues *Phe31, His32,* and *Trp118* and the benzene atoms of the TXL molecule's benzene core *A* involved in the hydrophobic interaction was 4.726 Å. The average distance between the nonpolar radical of the amino acid residue *Leu110* and the benzene core *B* atoms of the TXL molecule was 4.397 Å. Additionally, the formation of hydrogen bonds between the hydrogen atoms of the hydroxyl groups on benzene core *B* of the TXL molecule and the oxygen of the carbonyl group in *Leu110* was observed (Fig. 3B). The obtained data indicate the presence of non-covalent binding between the TXL molecule and the amino acid residues in the hydrophobic cluster 2 of the *α*-La molecule. Many studies have shown that non-covalent interactions are primarily responsible for the formation of stable polyphenol-protein complexes, significantly enhancing the bioavailability and absorption of polyphenols (Li et al., 2021; Shahidi & Dissanayaka, 2023).

Moreover, the mutual arrangement and conformation of the molecules facilitate electrostatic interactions between the negatively charged oxygen atom of the three‑carbon fragment of the TXL molecule and the positively charged amino group of *Lys114* in the protein molecule. It should be noted, however, that at pH values 5.5–6.8, which are typical for the most widely used WPCs, the TXL molecule carries a charge close to neutral (Das et al., 2021; Teixeira et al., 2005). Therefore, the electrostatic interaction identified in our study likely plays a less significant role in the binding of the polyphenol to the *α*-La molecule compared to hydrophobic and hydrogen bonds.

Thus, the placement of the TXL molecule within the recess containing the hydrophobic cluster of nonpolar amino acid residues on the surface of the *α*-La molecule, the correspondence of their sizes, and the nature and types of bonds formed suggest a high degree of complementarity. This indicates the formation of a stable *α*-La-TXL molecular complex.

#### 3.1.4 Prediction of interactions of TXL molecules with β-Lg and α-La under-heat treatment conditions

To enhance the interaction between proteins and polyphenols, most encapsulation methods involve heating (Li et al., 2023). Protein denaturation, induced by exposure to high temperatures, results in the opening of previously closed amino acid residues, thereby increasing the accessibility of binding sites and potentially facilitating the interaction with polyphenols (Le Maux, Bouhallab, Giblin, Brodkorb, & Croguennec, 2014). However, limited data are available regarding the interaction of polyphenols with *β-Lg and α-La* protein molecules under heat treatment conditions.

The interactions of TXL molecules with *β*-Lg and *α*-La were modelled using molecular dynamics simulations under heating conditions at a temperature of 80 °C. As a result of the applied thermal effect, conformational changes in the *β*-Lg molecule were observed, particularly at the *β*-loop site between sheets *C* and *D*, where intramolecular hydrogen bonds were formed between the amino acid residues *Lys60, Glu62,* and *Lys69*. These structural changes led to the formation of a new intermolecular hydrogen bond between the oxygen atom of the hydroxyl group on the benzene core *B* of the TXL molecule and the hydrogen of the amino group of *Lys69*. This suggests that the β-La-TXL molecular complex may exhibit enhanced stability under the employed conditions of thermal processing.

The involvement of the amino acid residues *Lys60, Glu62, and Lys69* in ligand binding, as identified in our study, aligns well with existing crystallographic data on the *β*-Lg complex with retinol palmitate (Wu et al., 1999).

In the *α*-La molecule, a reduction in the number of intramolecular hydrogen bonds was observed in the region of hydrophobic cluster 2, which may be attributed to the unfolding of protein structures as a result of thermal exposure (Brinkmann, Thiel, & Otzen, 2013; Permyakov, 2020). Simultaneously, only one intermolecular hydrogen bond was detected in the *α*-La-TXL system, formed between the hydrogen atom of the hydroxyl group of benzene core *B* of the TXL molecule and the oxygen of the carbonyl group of *Leu110*. These findings indicate that, unlike the *β*-La-TXL molecular complex, the *α*-La-TXL system may exhibit a reduced interaction strength between the polyphenol and protein molecules under the applied thermal processing conditions.

Upon modeling, no significant changes were detected in the number of atoms involved in the hydrophobic interaction or in the average distance between them for the *β*-Lg-TXL and *α*-La-TXL systems under the applied thermal conditions. Similarly, no substantial change in the distance between atoms engaged in the electrostatic interaction between TXL and *α*-La molecules was observed. It appears that the heating conditions employed are unlikely to markedly affect the strength of hydrophobic and electrostatic interactions in the molecular complexes formed between TXL and the major whey proteins.

It is well known that the proportion of *β*-Lg in whey is significantly higher than that of *α-*La (Madureira, Pereira, Gomes, Pintado, & Xavier Malcata, 2007). In light of this, the results of our molecular simulations suggest that under conditions of thermal treatment that induce denaturation of whey proteins, an increase in the efficiency of TXL encapsulation can be expected, owing to the formation of additional hydrogen bonds between the polyphenol and *β*-Lg molecules. Furthermore, it is important to note that protein denaturation may also intensify cross-linking of protein molecules into aggregates (Le Maux, Bouhallab, Giblin, Brodkorb, & Croguennec, 2014; Permyakov, 2020; Schong & Famelart, 2018; Volić et al., 2022), potentially enhancing the stability of the resulting intermolecular complexes (Chen, Remondetto, & Subirade, 2006; Ramos et al., 2017).

Based on the findings from our computer simulations, we conclude that both *β*-Lg and *α*-La molecules are capable of binding at least one TXL molecule each. Accordingly, in subsequent studies, the amount of polyphenol added to the liquid WPC was calculated based on their equimolar ratio.

### 3.2 Search for rational technological solutions for TXL encapsulation into whey proteins

In the first step, thermomechanical stirring of the reaction mixture was performed on a magnetic stirrer for 20 min, with heating to 80 ± 2 °C, to assess the efficiency of TXL encapsulation into whey proteins. The samples were then subjected to lyophilisation and subsequently pulverised.

TXL is known to have a bitter aftertaste. Organoleptic analysis revealed that after encapsulation using thermomechanical stirring under the specified conditions, the bitterness of the polyphenol was significantly reduced, though not entirely masked. These findings from the organoleptic analysis are consistent with the results of our spectrophotometric analysis (TXL encapsulation efficiency was 63 ± 3 %) and scanning electron microscopy.

**Fig. 4:**
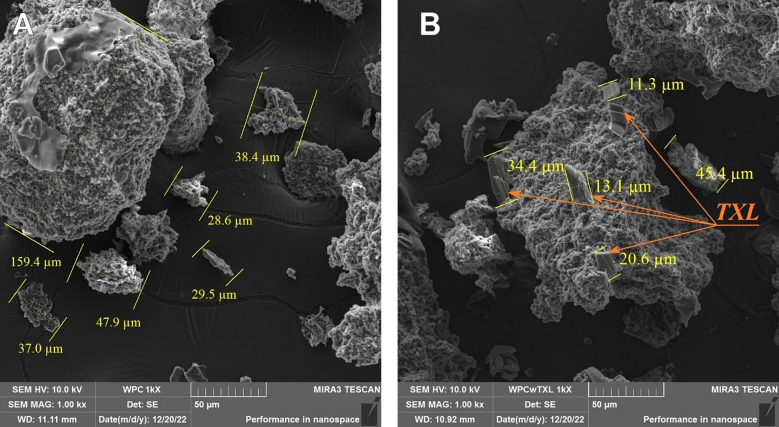
Scanning electron microscopy of lyophilised WPC obtained after thermomechanical stirring of the liquid concentrate in pure form (A) and with TXL (B).

Microphotographs of the dried WPC revealed that the thermomechanical stirring process led to the formation of whey protein aggregates up to 160 μm in size. Crystals of varying shapes and sizes were observed on the surface and within these aggregates in samples containing TXL (Fig. 4).

Contrary to the anticipated formation of equimolar complexes of whey proteins with polyphenol, the encapsulation method employed led to the formation of protein aggregates that fully or partially encapsulated large TXL crystals.

TXL is known to exhibit a tendency to polymerize, which occurs through the binding of its molecules via phenolic hydroxyl groups (Terekhov et al., 2019). Polymerization may lead to the formation of TXL crystals of varying sizes, which exhibit significantly reduced interactions with other substances and diminished biological activity compared to the monomeric form of the polyphenol (Fatkullin et al., 2021).

Thus, the well-established tendency of TXL molecules to crystallise may adversely affect both the extent of their encapsulation and the biological activity of the polyphenol.

Our results revealed that thermomechanical mixing does not fully address the challenges associated with encapsulating TXL in whey proteins. This method fails to form a continuous shell around the encapsulated material and does not effectively neutralize the bitter taste of the polyphenol. To enhance the efficiency of TXL encapsulation in whey proteins, it has been proposed to employ a method that ensures the micronisation of the interacting substances. This should promote the formation of monomeric TXL forms and ensure their full integration into the encapsulating material. One such technique, commonly utilised in the food industry, is spray drying (Banjare et al., 2019; Nesterenko, Alric, Silvestre, & Durrieu, 2013). Previous studies on the use of spray drying for the encapsulation of polyphenols in various high molecular weight compounds indicate its promising potential (Castro-López et al., 2021; Ćujić-Nikolić et al., 2019; Kulandaivelu & Mandal, 2017).

### 3.3 Analysis of encapsulation efficiency of spray-dried samples

It is well-established that whey protein denaturation occurs during drying, with the degree of denaturation increasing as the drying temperature in the chamber rises (Bernard et al., 2011). High temperatures reduce the solubility of proteins and impair the technological properties of the resulting dry protein concentrates (Amdadul Haque & Adhikari, 2014; Chegini et al., 2014). Consequently, our experimental studies aimed to evaluate the effect of several milder drying temperature regimes on the efficiency of TXL encapsulation into whey proteins. Spray drying was conducted at incoming air temperatures of 130, 150, and 170 °C.

**Fig. 5:**
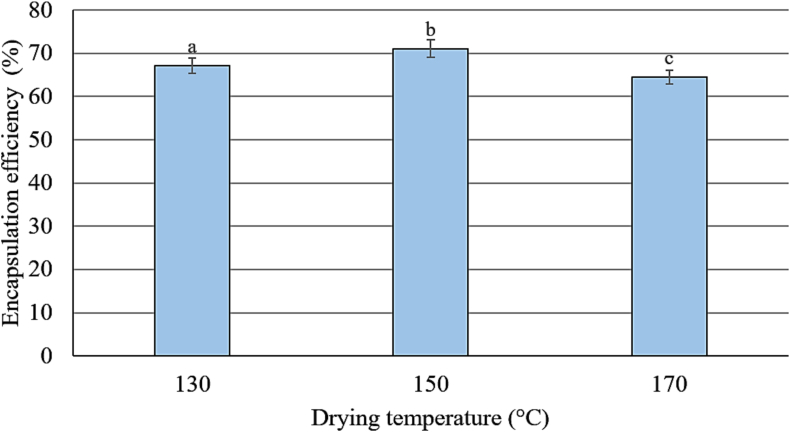
Efficiency of TXL encapsulation into whey proteins by spray drying at different inlet air temperatures. Error bars represent the standard deviation (SD) of the mean (*n* = 3). Different letters of the upper indices represent the difference at *p* < 0.05.

Organoleptic evaluation revealed that the taxifolin flavour was effectively masked regardless of the spray drying temperature regime. No bitterness was detected in any of the dry WPC samples. Spectrophotometric analysis indicated that the maximum encapsulation efficiency was achieved at 150 °C, reaching 71 ± 2 % (Fig. 5).

Fluorescence emission spectrometry is traditionally used to assess structural changes in proteins. Depending on the configuration of the polypeptide chain – specifically its secondary, tertiary, and quaternary structure – the orientation of aromatic amino acids, such as tryptophan, tyrosine and phenylalanine may vary. This variation in orientation can significantly affect the fluorescence emission intensity (Liang & Subirade, 2012; Shen et al., 2008). The *β*-Lg molecule contains two tryptophan residues, *Trp19* and *Trp61*, which are located in significantly different environments. *Trp19* is situated in an apolar environment within the cavity of the *β*-Lg molecule, while *Trp61* protrudes beyond the surface near a disulfide bridge formed by *Cys66* and *Cys160*, which can effectively quench its fluorescence. Therefore, the intrinsic fluorescence of *β*-Lg is predominantly attributed to *Trp19* (Palazolo et al., 2000).

**Fig. 6:**
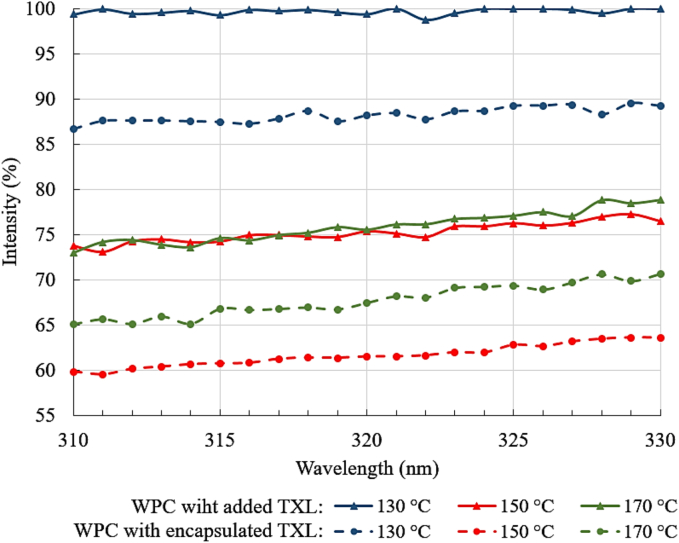
Fluorescence emission intensity of dry WPC obtained by spray drying at different temperature regimes with encapsulated or added TXL (% of fluorescence intensity of pure WPC).

A change in the configuration of the protein molecule, whether due to denaturation or complex formation with ligands, leads to alterations in the microenvironment of aromatic amino acids, which in turn affects fluorescence intensity (Liang & Subirade, 2012). Thus, the encapsulation of polyphenols traditionally uses fluorescence analysis to confirm the interaction between the protein and the phenolic molecule. The degree and strength of polyphenol binding can vary depending on the conditions, and the formation of a protein-polyphenol complex typically results in the screening of active fluorescence centres within the protein molecule, leading to fluorescence quenching (Liang & Subirade, 2012). The extent of quenching serves as an indicator of complex formation. In this study, to confirm the encapsulation, the quenching effect of encapsulated TXL was compared to an equal amount of TXL added directly to the WPC solution (WPC with added TXL) just prior to analysis (Fig. 6).

The results showed minimal changes in fluorescence intensity for dry WPC samples with added and encapsulated TXL obtained at a spray drying temperature of 130 °C. The addition of TXL during sample preparation had little or no effect on protein fluorescence (Fig. 6). TXL encapsulated at this drying temperature produced a weak quenching effect, with a decrease in fluorescence intensity of 10.5–12.7 %. This may be due to minimal changes in the configuration of the whey protein molecules under this milder drying regime.

Increasing the temperature in the drying chamber appeared to cause more significant changes in the configuration of protein molecules, thereby enhancing their interaction with TXL. At both 150 °C and 170 °C, spray drying induced more pronounced quenching of fluorescence, particularly in the samples with encapsulated TXL. The maximum decrease in fluorescence intensity was observed for dry WPC with encapsulated TXL obtained at 150 °C, where the fluorescence intensity decreased by 36.4–40.5 % compared to pure WPC and by 12.9–14.1 % relative to the sample with added TXL. This result is in agreement with the spectrophotometric data (Fig. 5), which showed the highest encapsulation efficiency at this drying temperature.

### 3.4 Shape and size analysis of whey protein polyphenol microcapsules using spray drying

**Fig. 7:**
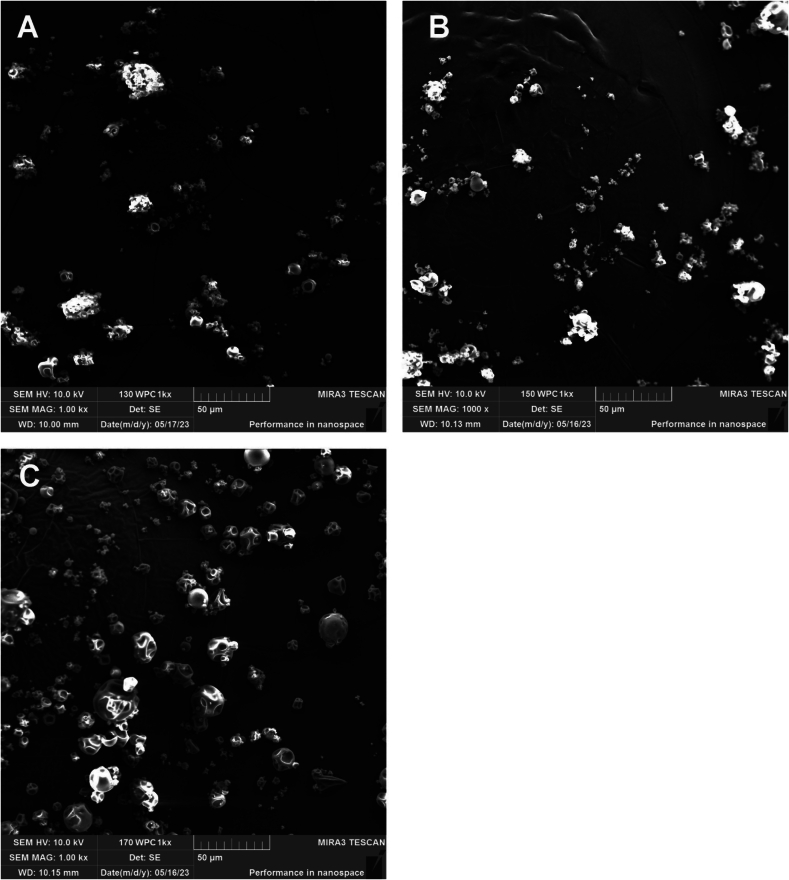
Scanning electron microscopy of TXL-encapsulated WPC obtained by spray drying at 130 °C (A), 150 °C (B), and 170 °C (C).

According to scanning electron microscopy data, the spray-dried WPC microcapsules containing TXL range in size from 3 to 30 μm. These microcapsules exhibit an irregular spherical shape, with visible depressions on the surface, and no apparent TXL crystals are visible (Fig. 7). The application of different drying temperature regimes (130, 150, and 170 °C) does not significantly affect the shape or size of the microcapsules. However, at the highest temperature of 170 °C, there is a slight increase in the proportion of larger microcapsules (18–30 μm in size).

**Fig. 8:**
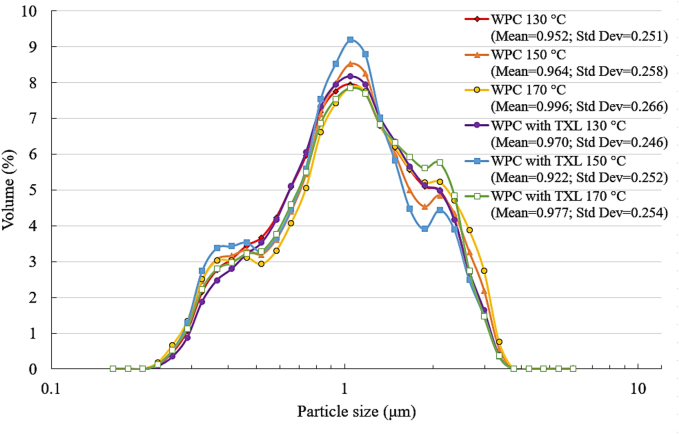
Particle size distribution of pure and TXL-encapsulated WPC obtained by spray drying at different temperature regimes.

Particle size analysis revealed that the average size of the particles within the WPC microcapsules does not change significantly with the incorporation of TXL and is not notably influenced by the spray drying temperature regime (Fig. 8).

The particle sizes of all the investigated WPC samples fall within the range of 0.2–3.7 μm, which is consistent with the findings of other studies (Both et al., 2020). These results indicate the absence of large TXL crystals within the spray-dried WPC microcapsules.

Thus, the microcapsules produced by spray drying are significantly smaller and more spherical in shape compared to those obtained by thermomechanical agitation and subsequent lyophilisation. Importantly, the microcapsules do not contain large TXL crystals, which aligns with the organoleptic evaluation. The bitterness of the polyphenol is completely absent when encapsulated using spray drying.

### 3.5 Antioxidant activity of TXL encapsulated in whey proteins using spray drying

The antioxidant activity of polyphenols was the primary characteristic that first drew the attention of scientists to this group of biologically active compounds (Pietta, 2000; Rice-Evans, 2012). Many of the beneficial effects of polyphenols on the human body are largely attributed to their ability to chelate free radicals (Das et al., 2021). As a result, assessing the antioxidant activity of TXL after its encapsulation in whey proteins and subsequent release is of significant importance.

Our studies revealed that dry WPC, in the absence of polyphenols, exhibited almost no antioxidant activity. However, when TXL was encapsulated into whey proteins by spray drying, no significant differences in antioxidant activity were observed across the temperature regimes tested. This could be due to the partial destruction of protein microcapsules during exposure to the aqueous ethanol solution used in sample preparation. Ethanol is known to denature proteins by disrupting intramolecular hydrogen bonds (Sato et al., 2000), with the degree of denaturation increasing with prolonged exposure (Nikolaidis & Moschakis, 2018). To address this, we evaluated the antioxidant activity at different exposure durations in a 60 % aqueous ethanol solution – short-term (1 min) and longer (20 min) (Table 1).

**Table 1:** Antioxidant activity of TXL in pure form and as part of spray-dried WPC microcapsules.

Dry matter	% of ABTS^●+^ inhibition	TEAC, μmol/g
TXL	12.93 ± 1.15 ^a^	25.86 ± 2.30 ^a^
WPC with TXL (exposure in 60 % aqueous ethanol solution 1 min)	8.47 ± 0.88 ^b^	16.94 ± 1.76 ^b^
WPC with TXL (exposure in 60 % aqueous ethanol solution 20 min)	11.50 ± 0.95 ^a^	23.00 ± 1.90 ^a^

Results are reported as mean values ± standard deviation (SD), *n* = 3. The mean values in each column followed by indices with different letters differed significantly (*p* < 0.05).

According to the obtained data, encapsulation of TXL reduces its antioxidant activity, which is probably explained by shielding of active centers of polyphenol molecules when forming complexes with whey proteins (Arts et al., 2001; Lopes et al., 2020; Zorilla et al., 2011). This decrease in the antioxidant activity of TXL agrees well with the results of our molecular modeling and may be due to the formation of hydrophobic and hydrogen bonds of its molecules with proteins.

Upon prolonged exposure of WPC with encapsulated TXL in 60 % aqueous ethanol solution, a recovery of the antioxidant activity of the polyphenol was observed (Table 1). The level of ABTS^●+^ inhibition increased from 8.47 % (when briefly incubated in aqueous ethanol solution) to 11.50 %. In TEAC units, this level corresponds to an antioxidant activity value of 23.00 μmol/g dry matter, while the value for pure polyphenol is 25.86 μmol/g.

Prolonged exposure to ethanol appears to lead to microcapsule disruption due to additional denaturation of whey proteins and release of bound polyphenol molecules. Thus, the antioxidant activity of TXL encapsulated in whey proteins by spray drying method, when they are ingested with food, can be fully manifested in the intestine after protein breakdown, which is consistent with available studies (Fang et al., 2011; Rohn et al., 2004).

## 4 Conclusions

The results of computer modeling enabled the prediction of the possibility of TXL encapsulation into whey proteins and revealed the intermolecular mechanisms underlying the formation of these complexes. A stable interaction between the TXL molecule model and the amino acid residues of the hydrophobic barrel of the *β*-Lg molecule, as well as residues Phe31, *His32*, *Leu110,* and *Trp118* within a small depression on the surface of the *α*-LA molecule, was established.

The thermomechanical stirring method used for encapsulation of TXL into liquid WPC at 80 ± 2 °C did not result in the formation of equimolar protein–polyphenol complexes. Instead, aggregates of protein molecules up to 160 μm in size were formed, which completely or partially encapsulated large TXL crystals. These complexes did not provide high encapsulation efficiency, which was found to be 63 ± 3 % according to spectrophotometric analysis, and they did not completely mask the bitterness of TXL.

When TXL was encapsulated in whey proteins using spray drying, the maximum encapsulation efficiency of 71 ± 2 % was achieved, as confirmed by Folin-Ciocalteu assay and fluorescence emission spectrometry. This was achieved with an inlet air temperature of 150 °C in the drying chamber. The size of the resulting WPC microcapsules ranged from 3 to 30 μm and showed little variation depending on the drying temperature regime used. Scanning electron microscopy revealed that the protein microcapsules had an irregular spherical shape, and no visible TXL crystals were found on their surface. These spray-dried microcapsules were characterised by the complete absence of the bitterness typically associated with TXL.

Regarding the antioxidant activity of encapsulated TXL, a decrease in its activity was observed after encapsulation by spray drying. The inhibition of ABTS^●+^ decreased from 12.93 ± 1.15 % to 8.47 ± 0.88 %. This reduction is likely due to the shielding of active centres of the polyphenol molecules as they form complexes with whey proteins. However, after incubating the dry WPC with encapsulated TXL in a 60 % aqueous ethanol solution for 20 min, the antioxidant activity of TXL showed a reversal in its dynamics. The level of ABTS^●+^ inhibition increased to 11.50 ± 0.95 %. This suggests that ethanol exposure disrupts the WPC microcapsules, releasing the bound polyphenol molecules. This finding indicates that when such WPC microcapsules are ingested with food, the antioxidant activity of TXL could be fully realised in the intestine after the breakdown of the protein structure.

Thus, spray drying can be regarded as a promising method for the encapsulation of TXL in whey proteins. This approach ensures the micronization of the interacting substances, provides a sufficiently high stability of the resulting complexes, offers protection for the antioxidant properties of the polyphenol against aggressive environmental conditions, and effectively masks its bitter taste.

The effectiveness of the proposed approach for the delivery of polyphenol molecules to specific sections of the intestine can be assessed through additional studies, including the analysis of the release processes of TXL from WPC microcapsules using an in vitro digestion model.

## CRediT authorship contribution statement

**Aleksander A. Borisenko:** Writing – original draft, Visualization, Methodology, Investigation, Conceptualization. **Tatyana N. Bobrysheva:** Writing – original draft, Validation, Methodology, Investigation, Conceptualization. **Marina S. Zolotoreva:** Writing – review & editing, Methodology, Investigation, Conceptualization. **Georgiy S. Anisimov:** Validation, Supervision, Project administration, Funding acquisition, Conceptualization. **Aleksey A. Borisenko:** Writing – review & editing, Methodology, Investigation, Conceptualization. **Dmitry G. Alexeev:** Investigation, Conceptualization. **Victoria G. Razinkova:** Visualization, Investigation. **Ekaterina G. Greseva:** Visualization, Investigation. **Svetlana S. Avanesyan:** Methodology, Investigation. **Marina N. Sizonenko:** Methodology, Investigation. **Natasa Poklar Ulrih:** Writing – review & editing, Supervision. **Itai S. Dzinamarira:** Investigation, Formal analysis.

## Declaration of competing interest

The authors declare that they have no known competing financial interests or personal relationships that could have appeared to influence the work reported in this paper.

## Data Availability

Data will be made available on request.
